# Diagnosis and treatment of Rasmussen's encephalitis pose a big challenge: Two case reports and literature review

**DOI:** 10.1016/j.amsu.2021.102606

**Published:** 2021-08-04

**Authors:** Ali Hammed, Maysaa Badour, Sameer Baqla, Fatema Amer

**Affiliations:** a Tishreen University Hospital, Department of Neurosurgery, Lattakia, Syria; b Pediatric University Hospital, Division of Neurology, Damascus, Syria

**Keywords:** Rasmussen encephalitis, Focal seizures, Epilepsia partialis continua, Cortical atrophy, Hemispherectomy

## Abstract

Rasmussen encephalitis (RE) is a rare disease of unknown etiology that causes severe chronic unihemispheric inflammatory disease of the central nervous system mainly in children. It leads to intractable seizures, cognitive decline and progressive neurological deficits in the affected hemisphere.

We report two cases of RE, as defined by fulfillment of the 2005 Bien criteria. The diagnostic challenge of characterizing this rare disease will be highlighted by the extensive serum, CSF, MR imaging and EEG data in the two patients. In addition, we will review the various forms of therapy attempted in these two patients, namely anti-epileptic drug therapy and immunomodulatory therapy. Hemispherectomy was done for the second patient with favorable outcomes of controlling seizures, but unfortunately, he died because of meningitis.

Until the causes of Rasmussen's encephalitis are known, it is difficult to anticipate how treatments will improve. Such a situation creates a therapeutic dilemma; hemispherectomy is not favored because of the inevitable postoperative functional deficits, but a real risk exists that treatments used to delay progression of the disease will defer definitive surgical treatment beyond the time when an optimum post-hemispherectomy outcome could be expected.

## Highlights

• **R**asmussen encephalitis (RE) causes severe chronic unihemispheric inflammatory disease.
• RE should be suspected in any patient with refractory seizures.
• Its recognition is important because early intervention with surgery can improve outcomes.

## 1 Introduction

**R**asmussen encephalitis (RE) is a rare disease of unknown etiology that causes severe chronic unihemispheric inflammatory disease of the central nervous system mainly in children. It leads to intractable seizures, cognitive decline and progressive neurological deficits in the affected hemisphere [1].

It is characterized by unilateral hemispheric atrophy, focal intractable seizures, and worsening neurological deficits.

The pathogenesis of RE has long been suspected to represent the result of adaptive immunity gone awry. Evidence has mounted in support of a cell-mediated hypothesis. Most of the infiltrating lymphocytesin RE are cytotoxic T-cells, and granzyme B-containing CD8þ cells may be seen apposed to MHC class I positive neurons and astrocytes [2,3].

The key point of the clinical syndrome of Rasmussen is the continuous partial seizures. Three stages through which the disease develops are currently recognized; initially it begins with a prodrome with sporadic and hemiparesis crisis; next is the acute stage where crises are increasingly frequent addition onset of neurological impairment, as cognitive disorder, hemiparesis, hemianopia and aphasia (if the dominant hemisphere is affected) thereafter patients spend a residual phase where already they established permanent neurological deficits and crises, although less frequently than in the acute phase; although some patients will remain hemiplegic [4].

RES now is termed as Rasmussen's encephalitis (RE). The disease typically begins in childhood with refractory focal motor seizures which gradually evolve into epilepsia partialis continua (EPC) which is followed by progressive hemiparesis and mental deterioration [5,6].

To date, there is no definitive consensus on treatment, with proposed strategies ranging from acute and chronic immunotherapy to hemispherectomy. Our two cases of RE exemplify the diagnostic and therapeutic challenges.

Treatment in Rasmussen's encephalitis aims to reduce seizure severity and frequency and improve the functional long-term outcome, as measured by both motor and cognitive performance. However, to date, treatments have only alleviated the symptoms65 and have not tackled the underlying causes [7].

This work has been reported in line with the SCARE criteria [16].

## 2 Case presentation

### 2.1 Case 1

An 8 year-old girl presented with history of repeated episodes of clonic seizures involving right upper and lower limbs and each episode lasting for 2–5 minutes with prolonged postictal state. The episodes didn't involve a sudden loss of consciousness but she had a bladder incontinence. The first episode started at the age of 7 years with rapid increase in seizure activity followed by decrease in frequency 10–15 episodes in the day with slurring of speech, cognitive delay and abnormal gait. She was born as a second twin and cried immediately after birth with normal APGAR score. No history of neonatal seizures. She had no history of blood transfusions, neurosurgery, traumatic head injury, proven allergies and family history of epilepsy. An initial routine electroencephalogram (EEG) was normal. Magnetic resonance imaging (MRI) was normal. The patient put on Oxcarbazepine and then, Valproate Sodium and Clonazepam were added gradually.

One month ago, she suffered from paraesthesia and twitching of right arm.

She admitted to our neurology department when the patient experienced an exacerbation of focal status epilepticus and generalized seizures.

Her seizures could not be controlled with antiepileptic drugs alone.

Neurologic exam at admission showed constant twitching of the right hand and intermittent stiffening of the right leg and pelvic. She had a right hemiparesis and muscle power Medical Research Council (MRC) grade 3 on the right upper and lower limb, but a full 5 on the left upper and lower limbs.

She also had exaggerated tendon reflexes on the right side. She showed severe global developmental delay.

There were no signs of meningeal irritation, and cranial nerves were intact. Cerebrospinal fluid analysis was normal, (PCR) for Herpes Simplex Virus, EBV, measles, Rubella and CMV appeared to be negative.

EEG showed slow activity with focal left frontal biphasic spikes paroxysmal discharges (Fig. 3).

MRI brain T1 revealed left hemispheric atrophy and in the left insular cortex (Fig. 2).

**Fig. 1:**
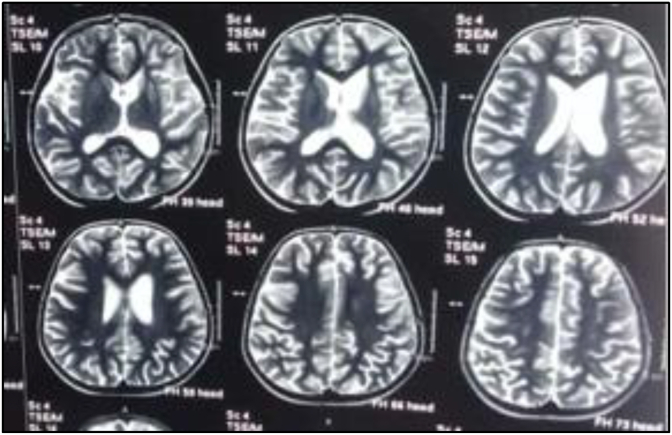
MRI T2 axial reveals atrophy in left Caudate and putamen nuclei.

MRI T2 axial revealed atrophy in left Caudate and putamen nuclei (Fig. 1).

**Fig. 2:**
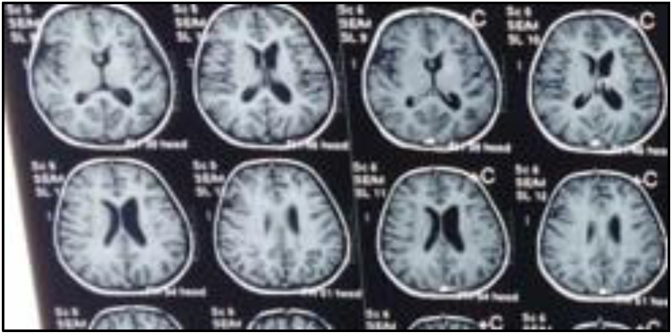
T1 shows atrophy in left insular cortex.

**Fig. 3:**
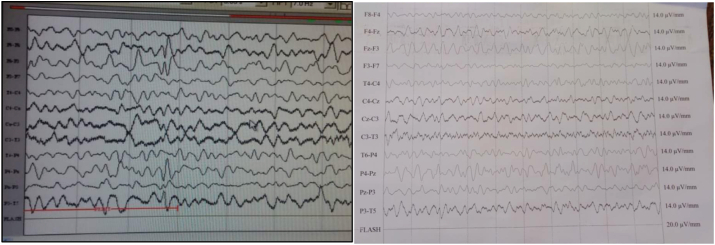
EEG shows slow activity with focal left frontal biphasic spikes paroxysmal discharges.

A diagnosis of RE was suspected.

She was given Immunotherapy with IVIG for one dose followed by IV. methyl prednisolone 30mg/kg for 5 days. Followed by oral prednisolone for 28 days. She slightly improved.

The parents however noticed an increase in the frequency of her seizure to three times a day every day. She is currently on outpatient review, and a more aggressive treatment (including surgical intervention) is being considered.

### 2.2 Case 2

A 5 year old male child presented with complaints of multiple episodes of clonic seizures of the left upper and lower limb since the last 8 months, each episode was lasting for 5–15 min with a prolonged postictal state. He was tre ated with anticonvulsant drugs. He had no history of blood transfusions, neurosurgery, traumatic head injury, proven allergies, and family history of epilepsy. Antenatal, perinatal and postnatal history was normal. The seizures were progressively increasing in frequency and severity since last month. The episodes also involved a sudden loss of consciousness and bladder incontinence. Because of that, he admitted to our neurology department. On examination, the child was disoriented. His vitals were stable. Central nervous system examination showed left-sided hemiparesis with exaggerated tendon reflexes on the left side. Muscle power Medical Research Council (MRC) was grade 4 on the left upper and lower limb, but a full 5 on the right upper and lower limbs. There were no signs of meningeal irritation, and cranial nerves were intact. Cerebrospinal fluid analysis was normal, (PCR) for Herpes Simplex Virus, EBV, measles and CMV, appeared to be negative.

The patient had a progression to epilepsia partialis continua within 9 months after first focal seizure and impairment in cortical functioning.

**Fig. 4:**
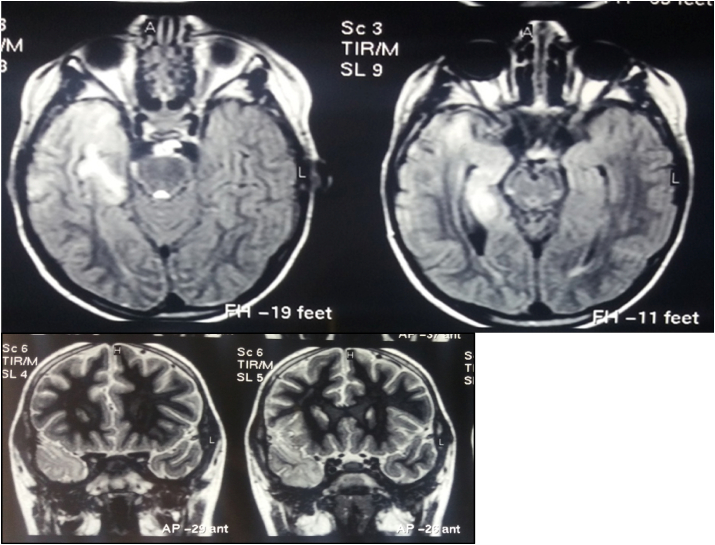
A: MRI Axial Flair T1, B: Coronal T2: Show high signal intensity area in right temporal lobe and Hippocampus.

The MRI Flair showed high signal intensity area in right temporal lobe (Fig. 4).

**Fig. 5:**
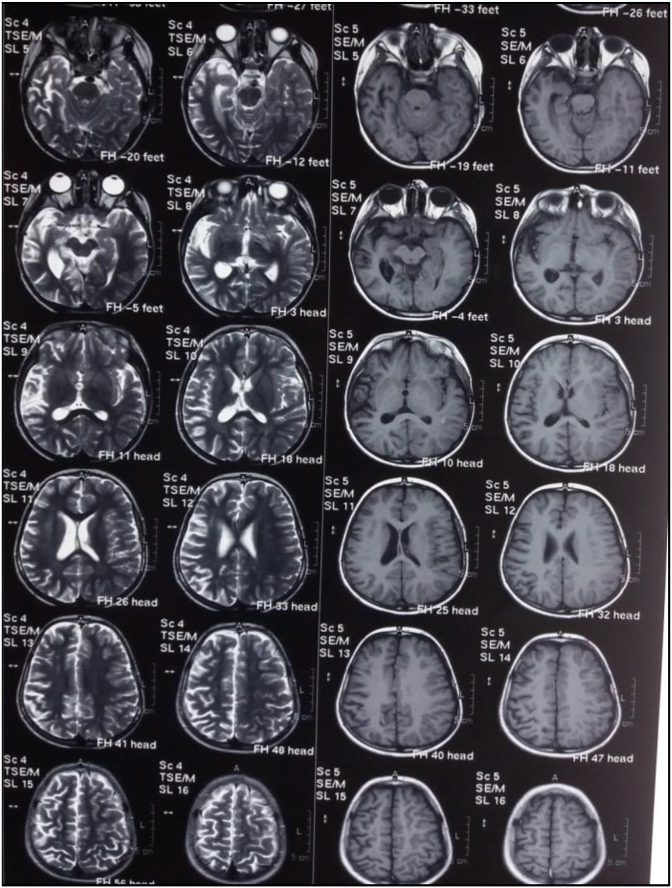
MRI T1 (B) and T2 (A)(Axial) show atrophy in right partial and temporal lobes.

MRI T1 and T2 showed atrophy in right partial and temporal lobes (Fig. 5).

**Fig. 6:**
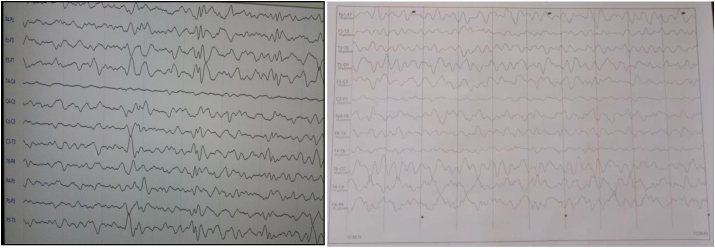
EEG shows slow activity with focal right frontal polyphasic spikes paroxysmal discharges.

EEG showed slow activity with focal right frontal polyphasic spikes paroxysmal discharges (Fig. 6).

On arrival he was give diazepam and phenytoin infusion. Later treatment was started on oral levetiracetam, clonazepam, lamotrigin, Carbamzepine and valproate sodium.

After 25 days of treatment, patient continued to develop seizures and her right-sided weakness persisted, progressed and became worsen. During these episodes of fits, child was started on intravenous steroids.

To slow hemispheric atrophy, anti-inflammatory treatment with tacrolimus was given.

Follow up MRI revealed effacement of cortical sulci and also asymmetry between two cerebral hemisphere with atrophy of right cerebral hemisphere.

The patient was treated with regular dosing of IVIG. A dose interval of 4 weeks was determined. Despite regular IVIG dosing for approximately 6 months as well as concomitant AED therapy, the patient's epilepsy remained refractory. Functional Hemispherectomy was performed by the complete removal of the cortex and white matter, sparing the basal ganglia. After surgery, the child had mild episodes of seizures. Unfortunately, he died because of meningitis.

## 3 Discussion

Rasmussen's encephalitis was first described by neurosurgeon Theodore Rasmussen and his colleagues in the late 1950s [1].

Since then, the variable clinical features and lack of understanding of cause have created dilemmas in clinical decision making. The 2005 European consensus on pathogenesis, diagnosis, and treatment of Rasmussen's encephalitis remains the accepted guideline for evaluative criteria. The clinical diagnostic criteria for RE were proposed by Derry et al. (Table 1) [8,9].

**Table 1:** Diagnostic criteria for Rasmussen's encephalitis. Rasmussen's encephalitis can be diagnosed if either of the three criteria in part A or two out of three criteria of part B are present. Check first for the features of part A. In addition, if no biopsy is performed, magnetic resonance imaging with administration of gadolinium and cranial computerized tomography need to be performed to document the absence of gadolinium enhancement and calcifications to exclude the differential diagnosis of an unihemispheric vacuities ((Derry et al., 2002).

Part A	1. Clinical focal seizures (with or without epilepsia partialis continua) and unilateral cortical deficits 2. EEG: Unihemispheric slowing with or without epileptiform activity and unilateral seizure onset 3. MRI: Unihemispheric focal cortical atrophy and at least one of the following: Grey or white matter T2/FLAIR hyperintense signal Hyperintense signal or atrophy of the ipsilateral caudate head
Part B	1. Clinical epilepsia partialis continua or progressive* unilateral cortical deficit(s) 2. MRI progressive* unihemispheric focal cortical atrophy 3. Histopathology T cell dominated encephalitis with activated microglial cells (typically, but not necessarily forming nodules) and reactive astrogliosis. Numerous parenchymal macrophages, B cells or plasma cells or viral inclusion bodies exclude the diagnosis of RE

MRI: Magnetic resonance imaging, RE: Rasmussen's encephalitis, EEG: Electroencephalograph.

٭ Progressive’ means that at least two sequential clinical examinations or MRI studies are required to meet the respective criteria. To indicate clinical progression, each of these examinations must document a neurological deficit, and this must increase over time. To indicate progressive hemiatrophy, each of these MRIs must show hemiatrophy, and this must increase over time.

In our two patients, all the three criteria were present from part A, which comprises focal seizures with EPC “epilepsia partialis continua " (EPC was found only in second patient) and unilateral cortical deficits in the form of hemiplegia, unihemispheric EEG slowing, and focal cortical atrophy with atrophy of ipsilateral caudate nucleus in brain MRI. Hence, the diagnosis of RE was suspected. The differential diagnosis of stroke, cerebral vasculitis, multiple sclerosis, Creutzfeldt-Jakob disease and subacute sclerosing panencephalitis were considered, but were ruled out on the basis of history, laboratory investigations, and the absence of associated characteristics in MRI and EEG findings.

Some authors state that in the absence of histopathological evidence, MRI with administration of gadolinium and cranial CT must be performed to evaluate for enhancement and calcifications and exclude vasculopathy (e.g., Sturge–Weber syndrome) [8].

Only a histopathologically demonstrated vasculitis of the type described by Derry and colleagues in one single case could be mistaken for RE on the basis of these criteria without brain biopsy [8,9].

Although the pathologic findings often resemble that of a viral encephalitis, attempts at identifying a viral etiology have been mixed and reliable identification of an offending infectious agent has not been successful. Given the presence of autoantibodies in many cases, particularly GluR3 autoantibodies, a variety of immunotherapy treatments have been attempted with varied success (Prayson et al., 2012) [10].

Rasmussen's encephalitis is now believed to be an ongoing and progressive immune-mediated process which induces apoptotic neuronal cell death and involves the neuroglial and lymphocytic response, leading to progressive deterioration of a single hemisphere [2,9].

Geller et al.12 describe a patient with an initially normal MRI and clinical course that progressed to epilepsia partialis continua within three months. Follow-up MRI done after development of epilepsia partialis continua showed atrophy. Kim et al.13 also showed that atrophy is more likely to be present in Rasmussen's encephalitis patients after the onset of epilepsia partialis continua [5,11].

Our first patient was found to have progression from her first seizure to epilepsia partialis continua within 9 months, impairment in cortical functioning (a graphesthesia) and decline in motor functions very early (within 8 months of onset) without signs of atrophy on imaging. This time course is quite unique upon review of the descriptions of Rasmussen's encephalitis in the literature, and it is even more exceptional to have an MRI without atrophy in her clinical state.

It remains uncertain whether the inciting antigens in this cell-mediated attack are endogenous or reflect an as yet undiscovered pathogen (e.g., cryptic viral infection). Why such an attack would target the cortical and subcortical structures of only one hemisphere and the predilection for such an attack to occur in early childhood remain a mystery.

Seizures are typically medically intractable or become so over time, and the goal of seizure control has to be tempered by overall quality-of-life considerations. Immunosuppressive and immunomodulatory therapies (e.g., tacrolimus) may slow disease progression.

Complete surgical hemispheric resection (hemispherectomy) and hemispheric disconnection (HD, functional hemispherotomy) are the only established methods to cure seizures in RE (success rates of 70–80 %) [12].

Hemispherectomy was done for the second patient with favorable outcomes of controlling seizures, but unfortunately, he died because of meningitis.

Until the causes of Rasmussen's encephalitis are known, it is difficult to anticipate how treatments will improve. Various attempts using immunotherapy have been tried in the past decade. Some slow the progress of the disease, but none has successfully cured or even halted the progression of disease. Such a situation creates a therapeutic dilemma; hemispherectomy is not favored because of the inevitable postoperative functional deficits, but a real risk exists that treatments used to delay progression of the disease will delay definitive surgical treatment beyond the time when an optimum post-hemispherectomy outcome could be expected [13].

The diagnosis of RE rests on clinical, electrophysiological (EEG) and morphological studies (MRI, in some cases histopathology). In most chronic patients (i.e. after a disease duration of >1 year), differential diagnoses are few. The particular challenge, however, is the early recognition of the disease, i.e. before progressive hemiatrophy and progressive loss of neurological functions is evident. Early diagnosis is desirable (Bien et al., 2002*c*,*d*; Granata et al., 2003*b*) as immunosuppressive therapy may be most effective at this time [14,15].

## 4 Conclusion

RE is rare disease entity and should be suspected in any patient with refractory seizures and progressive unihemispheric cortical atrophy with motor deficit. Its recognition is important because early and timely intervention with surgery can improve patient outcomes.

## Sources of funding

This research did not receive any specific grant from funding agencies in the public, commercial, or not-for-profit sectors.

## Ethical approval

This two case reports didn't require review by Ethics committee, Tishreen university hospital, tishreen university, Faculty of medicine, Lattakia-Syria.

## Registration of research studies

The case report at hand is not a first-in-man case report of a novel technology or surgical technique, therefore a registration of these case reports according to Declaration of Helsinki 2013 is not required.

## Consent for publication

Written informed consent was obtained from the patients' parents for publication of these two case reports and accompanying images. A copy of the written consent is available for review by the Editor-in-Chief of this journal.

## Author contribution

Dr. Ali Hammed (corresponding author): Contribution to the paper: first author, data collection, data analysis and interpretation, writing the paper. Maysaa Badour: Contribution to the paper. Writing Case Presentation. Dr. Sameer Baqla: Contribution to the paper: Treatment and examination of the patient, Writing the paper. Dr. Fatema Amer: Contribution to the paper: Writing the paper.

## Guarantor

Ali Hammed.

## Declaration of competing interest

The authors declare that they have no known competing financial interests or personal relationships that could have appeared to influence the work reported in this paper.
